# Improving Primary Healthcare for Elderly Patients: How Chronic Disease Management Intensity Makes a Difference

**DOI:** 10.34172/ijhpm.9152

**Published:** 2026-03-10

**Authors:** Jia Peng, Di Liang, Shailendra Prasad, Sumit Kane, Weijun Zhang, Yuxia Wu, Jiayan Huang, Yongsong Luo, Yin Dong

**Affiliations:** ^1^School of Public Health, Shanghai Institute of Infectious Disease and Biosecurity, Fudan University, Shanghai, China.; ^2^NHC Key Laboratory of Health Technology Assessment, Fudan University, Shanghai, China.; ^3^Center for Global Health and Social Responsibility, University of Minnesota, Minneapolis, MN, USA.; ^4^Nossal Institute for Global Health, The University of Melbourne, Melbourne, VIC, Australia.; ^5^David Geffen School of Medicine, University of California Los Angeles, Los Angeles, CA, USA.; ^6^Yichuan Street Community Health Service Center of Putuo District, Shanghai, China.; ^7^Health Commission of Yuhuan, Taizhou, Zhejiang Province, China.; ^8^The People’s Hospital of Yuhuan, Taizhou, China.

**Keywords:** Chronic Disease Management, Primary Healthcare, Length of Follow-up, Healthcare Utilization, Health Outcomes

## Abstract

**Background:**

Since 2009, China has implemented a chronic disease management program within primary healthcare (PHC) institutions in response to challenges posed by an aging population. However, the effectiveness of the program has been reported as mixed, likely due to variations in PHC physicians’ efforts and the support they received from the health system and community. This multi-sector engagement was conceptualized as management intensity in this study, and its impact on the program’s effectiveness was evaluated.

**Methods:**

This study analyzed 60 885 patients under the chronic disease management program in Yuhuan, Zhejiang province, as of 2023. Management intensity, the primary predictor, was quantified by township-level residual measured based on patients’ length of follow-up after eliminating patient demographics. This approach removed the portion of follow-up length attributable to individual characteristics, leaving the residual serving as a purified exposure variable for management intensity. The outcome measures included outpatient visits, inpatient admissions, outpatient and inpatient expenses, and glycemic and blood pressure (BP) control status. Data sources included chronic disease management registration records, service records of follow-up, and electronic medical records. A two-level mixed-effects regression model was then used to examine how management intensity affected the outcomes.

**Results:**

Each unit increase in management intensity corresponded to 0.21 more PHC outpatient visits and 0.15 fewer hospital outpatient visits. Meanwhile, higher management intensity was also associated with increased utilization of PHC inpatient services (odds ratio [OR]: 0.98, 95% CI: 0.97-0.98) and decreased utilization of hospital inpatient services (OR: 1.24, 95% CI: 1.18-1.29).

**Conclusion:**

Greater management intensity correlated with better health outcomes and higher utilization of PHC services. Since multi-sector engagement strongly affected how intensively chronic diseases were managed, it was imperative for health systems and communities to actively participate in and strengthen the program by supporting physicians.

## Introduction

**Key Messages**

**Implications for policy makers**

In the context of an aging population, the health system and the community should provide support for primary healthcare (PHC) physicians to facilitate their dedication to chronic disease management:

- Firstly, an integrated health information system aggregating residents’ health data could generate personalized treatment plans through artificial intelligence. This system provided substantial support for PHC physicians in managing the complex health conditions of elderly patients and simplified the physicians’ work.
- Secondly, performance-based bonuses and more opportunities for career advancement should be provided for PHC physicians, if they get higher scores in their key performance indicators.
- Thirdly, community workers could serve as a bridge between patients and PHC physicians, facilitating physicians’ understanding of elderly patients’ needs while also conveying health advice to the elderly in a clear manner.

**Implications for the public**

Against the backdrop of a rapidly aging population, the prevalence of chronic diseases continues to rise, posing increasingly severe challenges for prevention and control. Primary healthcare (PHC) serves as an effective approach to chronic disease management, particularly in low- and middle-income countries (LMICs). However, these countries still face multiple challenges in leveraging PHC services for chronic disease prevention and control. This study demonstrated that by strengthening the collaborative participation of PHC physicians, the health system, and the community in primary-level chronic disease management services, it was possible to enhance patients’ utilization of PHC services, reduce their reliance on hospital-based care, and ultimately improve health outcomes.

As populations worldwide continue to age, chronic non-communicable diseases (NCDs) have become a major global health challenge. Health systems frequently struggle to address NCDs effectively through their traditional reactive and episodic care models. However, patients with NCDs, especially the elderly, require person-centered, integrated care that involves long-term, continuous interaction with care providers.^1,2^ Such interaction enables providers to account for patients’ individual circumstances—including cognitive and physical impairments, low levels of education, and difficulty making lifestyle changes—and to provide targeted assessment and preventive, curative, and rehabilitative care.^3^ Primary healthcare (PHC), with its broad accessibility, cost-effectiveness, and community-based approach, has the potential to serve as a cornerstone for providing such person-centered, integrated care for patients with NCDs in the context of an aging population.^4,5^

 However, PHC institutions in low- and middle-income countries (LMICs) often face challenges in providing adequate care for patients with NCDs.^6^ This challenge is especially apparent in China. With basic medical insurance coverage in China surpassing 95%, financial barriers to accessing PHC services are negligible for most of the population. However, due to the absence of an effective gatekeeping system and widespread distrust in the quality of PHC services, patients in China tend to bypass primary care and seek services at higher-level healthcare institutions.^7^ In response, the Chinese government has implemented a chronic disease management program within PHC institutions since 2009, focusing on hypertension and diabetes.^8,9^ This program mandated that PHC institutions provide a comprehensive package of services to residents aged 35 and older within their designated regions. PHC physicians are responsible for screening eligible patients, enrolling them in the program with informed consent, and delivering ongoing, consistent follow-up care.

 While the chronic disease management program is a national policy issued by the Chinese government and implemented uniformly across the country, variations in the program’s effectiveness have been reported.^10,11^

**Figure 1:**
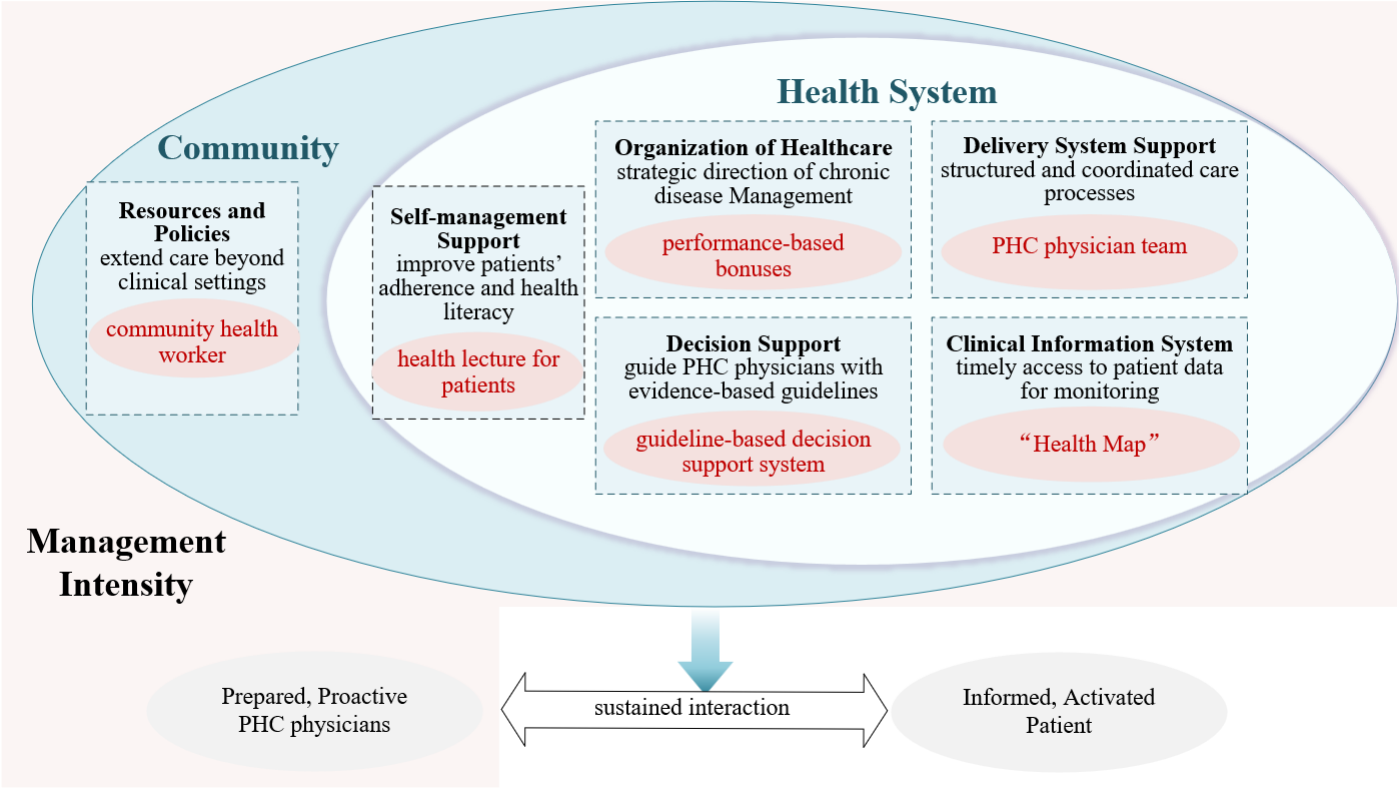
Model for Improvement of Chronic Disease Care. Notes: This figure was adapted from the Chronic Care Model proposed by Wagner in 1998. The text highlighted in red illustrated the support provided to PHC physicians by the health system and community in Yuhuan. Abbreviation: PHC, primary healthcare.

 Plausible explanations for these variations include the differences in the level of effort PHC physicians dedicate to chronic disease management and the extent of support physicians received from the health system and community. According to the Chronic Care Model,^12^ the health system and community can support PHC physicians and enhance chronic disease management through six interrelated components (Figure 1). For example, the *organization of healthcare* provides strategic direction of chronic disease management, the *delivery system support* ensures structured and coordinated chronic disease care processes, the *decision support* guides PHC physicians in service delivery with evidence-based recommendations, and the *clinical information system* enables timely access to patient data for monitoring and planning. These factors enable PHC physicians to respond effectively to the complexities of elderly patients, simplify physicians’ work, and incentivize their efforts in providing services, including systematic assessments, personalized treatment plans, and health education. Through the joint engagement of the health system, the community, and PHC physicians, patients experience high-quality care, which fosters their willingness to seek long-term chronic disease treatment and management in PHC settings.^13,14^

 Several studies have utilized management intensity to quantify the multi-sector engagement in chronic disease management.^15,16^ These studies suggested that higher management intensity was associated with lower healthcare costs and improved health outcomes.^15,17,18^ Most of these studies, however, have measured management intensity using the frequency of follow-up visits.^17-19^ While useful, this metric may not fully capture the management intensity, as patients lost to follow-up are excluded from ongoing care.^20^ More importantly, most previous studies were conducted in high-income countries and might not apply to China’s chronic disease management program, which requires PHC physicians to follow up with patients at fixed frequencies (eg, four times per year). Therefore, the length of follow-up could better reflect the strength of the physician-patient relationship, as well as the combined efforts of PHC physicians, the health system, and the community. However, none of the previous studies have considered the duration aspect of chronic disease management.

 Therefore, this study used the length of follow-up as a proxy for management intensity to explore its association with patients’ healthcare utilization, healthcare expenses, and health outcomes. Moreover, this study stratified patients into high-frequency and low-frequency follow-up groups and analyzed how management intensity affected each group.

## Methods

###  Study Design and Research Site

 This study conducted a cohort study to evaluate the impact of management intensity on the effectiveness of the chronic disease management program.

 The study was carried out in Yuhuan, a county located in Zhejiang province in south-eastern China. Yuhuan comprised 11 townships, including three urban and eight rural townships. As of 2023, the county had a resident population of 643 000, an aging rate of 15.40%, and a per capita gross domestic product of US$24 232 for registered residents. The county’s healthcare institutions included four public hospitals, ten township health centers, one community health center, and 175 village clinics. All of these institutions, except for the four public hospitals, were classified as PHC institutions.

 Since 2009, Yuhuan has implemented a chronic disease management program, providing diabetes and hypertension care through resident-based PHC physician teams. Faced with a rapidly aging population, Yuhuan has taken several measures under the framework of Chronic Care Model to enhance chronic disease management by supporting PHC physicians’ work. Specifically, the health system in Yuhuan developed an integrated health information system—Health Map—to enhance work efficiency. Moreover, performance-based bonuses were provided for PHC physicians to motivate their efforts. Meanwhile, the community in Yuhuan facilitated the appropriate involvement of community workers to assist PHC physicians in communicating with patients (Figure 1). By 2022, 90.47% of patients diagnosed with chronic diseases in Yuhuan were receiving chronic disease management from PHC physicians.

 To evaluate the impact of the multi-sector engagement on care effectiveness, the cohort for our study consisted of all patients enrolled in the chronic disease management program from 2009 to 2023. Follow-up visits were conducted to gather data on patients’ health outcomes and healthcare utilization over time.

###  Data Sources

**Figure 2:**
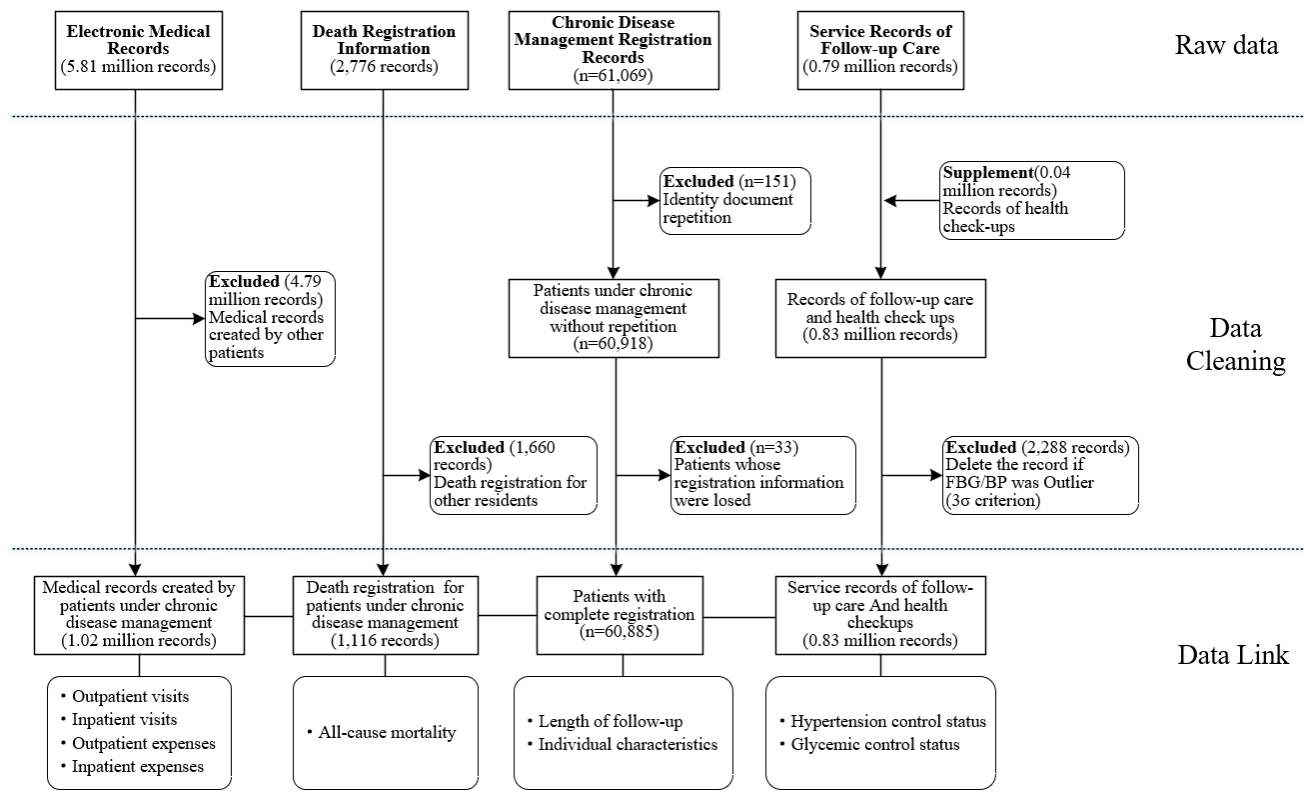
Data Cleaning Process. Abbreviations: FBG, fasting blood glucose; BP, blood pressure.

 Using identity documents, this study linked several databases, including chronic disease management registration records, service records of follow-up care, electronic medical records, and death registration information (Figure 2). The number of PHC physicians (per 1000 residents) in each township (2023) was also obtained from the Health Commission in Yuhuan. The data cleaning code is presented in Supplementary file 1.

####  Chronic Disease Management Registration Records

 The chronic disease management program included both diabetes and hypertension management programs. Patients were enrolled in either or both management programs. Each record corresponded to an individual. The registration records contained individual characteristics (age, gender, years of education, and residential address), years since diagnosis, and date of enrollment in the program. The data covered all patients undergoing chronic disease management between 2009 and 2023.

####  Service Records of Follow-up Care

 Each follow-up care for patients under management was automatically recorded in the service record database. The service record included the date of the follow-up, systolic blood pressure (SBP), diastolic blood pressure (DBP), and fasting blood glucose (FBG) measured at each follow-up care. With this information, we obtained the patients’ blood pressure (BP) and blood glucose in 2023. However, not all patients were followed up annually to have their BP and FBG measured. PHC institutions in Yuhuan provided health check-ups for residents, which included measurements of SBP, DBP, and FBG. Thus, patients’ health check-up data in 2023 were used as a supplement.

####  Electronic Medical Records Within the County

 The records of outpatient and inpatient visits were collected from the electronic medical records of public hospitals and PHC institutions located inside the county. Each record included the date of visits, the institution visited, and healthcare expenses. These cross-sectional data covered all patients who visited the aforementioned healthcare institutions in 2023. Patients under chronic disease management were identified through their identity documents.

####  Death Registration Information

 The death registration records of residents in Yuhuan for the year 2024 were obtained from the Centers for Disease Control and Prevention. Personal information was anonymized to protect privacy.

###  Measures

####  Outcome Variables

 This study designated healthcare utilization indicators as the primary outcome variables and health outcome indicators as the secondary outcome variables, all measured at the individual level.

 The variables employed to evaluate the healthcare utilization included outpatient visits, inpatient admissions, outpatient expenses, and inpatient expenses. Health outcomes were evaluated based on glycemic control status, BP control status, and all-cause mortality. Except for inpatient admission, glycemic control status, BP control status, and mortality, the others were all continuous variables.

- Outpatient visits: Number of outpatient visits to PHC institutions and hospitals in 2023.
- Inpatient admission: A binary variable indicating whether the patient was admitted as an inpatient in 2023 (1 if admitted, 0 otherwise), including admission to PHC institutions and hospitals.
- Outpatient expenses: Healthcare expenses incurred in PHC institutions and hospitals for outpatient visits during 2023.
- Inpatient expenses: Healthcare expenses incurred by a patient during 2023 for PHC inpatient admission or hospital inpatient admission.
- Glycemic control status: A binary variable indicating whether the patient’s average FBG was below 7.0 mmol/L in 2023 (1 if controlled, 0 otherwise).
- BP control status: A binary variable indicating whether SBP <140 mm Hg and DBP <90 mm Hg in 2023 (1 if controlled, 0 otherwise).
- All-cause mortality: A binary variable indicating whether the patient died in 2024 (1 if died, 0 otherwise).

####  Primary Predictor

 Management intensity, the primary predictor in this study, reflected the multi-sector engagement in chronic disease management. Empowered by the health system and community support, PHC physicians who exerted greater engagement could provide higher-quality, more patient-centered care. These superior services could attract and retain patients for ongoing chronic disease management within PHC. Therefore, the length of follow-up (from enrollment in the management program to the last follow-up) was used as the indicator to measure the management intensity of each township in Yuhuan.

 However, since individual factors such as age, gender, and educational level could impact the continuity of follow-up,^21,22^ the observed follow-up length could be conceptualized as comprising two components. One component was attributable to the multi-sector efforts of PHC physicians, the health system, and the community, which was explained as management intensity. Another component arose from patients’ individual factors. Therefore, employing unadjusted follow-up length would introduce measurement error into the estimation of management intensity and lead to issues of multicollinearity. To deal with these issues, this study applied a residual-based method to remove the components attributable to individual factors and mitigate multicollinearity.

 In the first step, the following regression was performed to obtain the residual, which represented the portion of follow-up length that could not be explained by patient demographics:


(1) $$t_{i}=X_{i}\beta +\eta_{i}$$

where *t_i_* was the length of follow-up (number of years) for patient *i* from enrollment to the last follow-up visit as of 2023. *Xi* included gender, age, years of education, years since diagnosis, age squared, years since diagnosis squared, indicators for enrollment in hypertension management and diabetes management for patient *i*, as well as the number of physicians in the township (per 1000 residents). The residual *η_i_* was orthogonal to individual factors. Consequently, the residual could serve as a purified exposure variable for management intensity, achieving smaller measurement error and avoiding multicollinearity. The regressions were conducted among patients who had been enrolled in the chronic disease management program as of 2023.

 In addition to performing the regression for patients in the entire sample, we also conducted the regression for the subsamples of diabetes patients and hypertension patients, separately. The entire sample included patients in either or both management programs. The subsample of diabetes patients included patients under diabetes management, regardless of a diagnosis of hypertension. The subsample of hypertension patients included patients under hypertension management, regardless of whether they were diabetes-managed patients. As shown in Figure S1, Supplementary file 1, the management intensity calculated in the subsample of diabetes patients and hypertension patients was highly correlated, since the services were provided by the same PHC institutions and physicians. Therefore, the management intensity calculated in the entire sample was adopted as the indicator to evaluate the management intensity. For the patients who were enrolled in both diabetes and hypertension management, the length of follow-up was calculated as the larger of either diabetes or hypertension management program length, and so was the number of years since diagnosis.

 In the next step, we took the mean of the individual residuals (*η_i_*)over each township *m* as the management intensity score *l_m_* for the townships:


(2) $$l_{m}=\frac{1}{n_{m}}\sum_{i\in m}\eta_{i}$$

where *n_m_* represented the number of patients under chronic disease management in township *m*. *l_m_* captured the effort that PHC institutions and physicians in township *m* have put into chronic disease management from 2009 to 2023. Townships that put more effort into chronic disease management had higher management intensity scores. Then we transformed the score (*l_m_*)into a rank variable (*PHCrank_m_*), which ranged from 1 to 11. The township with the lowest management intensity score had a *PHCrank_m_* of 1, and the highest had a *PHCrank_m_* of 11.

####  Control Variables

 Control variables in this study included gender, age, years of education, years since diagnosis, and the Charlson Comorbidity Index (CCI) for patient *i*. Many patients with chronic diseases often suffer from multiple comorbidities at the same time.^23^ In order to exclude the influence of comorbidities on the results, the CCI was included as a control variable in this study.^24^ A CCI for each patient was calculated based on the International Classification of Diseases, 10th Revision (ICD-10) in the records of outpatient and inpatient visits.^25^ Health information systems were widely used in PHC institutions across China. Since ICD-10 codes were designed as drop-down menus for physicians to select in the information system, the codes were reliable at the PHC level.^26^ The number of PHC physicians (per 1000 residents) in each township was also used as a control variable.

###  Statistical Analysis

####  Two-Level Mixed-Effects Linear Regression

 To address the problem of similarity within groups (village level), this study estimated two-level mixed-effects linear regressions to evaluate the impact of management intensity on patients’ healthcare utilization. The first level was the individual level, and the second level was the village level:


(3) $$Y_{ijm}=\beta_{0}+\beta_{1}PHCrank_{m}+X_{i}\beta_{2}+T_{m}\beta_{3}+u_{j}+\varepsilon_{ij}$$

where the dependent variable *Y_ijm_* was healthcare utilization at the individual level. It was explained by the following variables: outpatient visits (PHC and hospital), outpatient expenses (PHC and hospital), and inpatient expenses (PHC and hospital). All these variables were cross-sectional data in 2023. *PHCrank_m_* was the indicator of management intensity for township *m* according to the intensity score. *X_i_* included gender, age, years of education, years since diagnosis, and the CCI for patient *i*. *T_m_* was the number of physicians (per 1000 residents) within the township *m*. *u_j_* was the random effect at the village level.

 The regressions were conducted in the entire sample, the subsample of diabetes patients, and the subsample of hypertension patients. The regressions for inpatient expenses (including PHC inpatient expenses and hospital inpatient expenses) were conducted among patients who had inpatient records in 2023 and were under chronic disease management.

 In addition, we stratified patients based on the frequency of follow-up. According to the requirements of the related policy, PHC physicians were mandated to provide at least four follow-up visits per year for each patient. Therefore, this study categorized patients into a high-frequency group (≥4 follow-ups/year) and a low-frequency group (<4 follow-ups/year). The impact of management intensity on patients was then analyzed separately within these two groups.

####  Two-Level Mixed-Effects Logistic Regression

 For 0-1 variables, we employed two-level mixed-effects logistic regressions to explore the association between *PHCrank_m_* and these outcome variables. The first level was the individual level, and the second level was the village level:


(4) $$Logit\left(\frac{P(Y_{ijm}=1)}{P(Y_{ijm}=0)}\right)=\beta_{0}+\beta_{1}PHCrank_{m}+X_{i}\beta_{2}+T_{m}\beta_{3}+u_{j}+\varepsilon_{ij}$$

where *Y_ijm_* included the indicators of inpatient admission and indicators of health outcomes (glycemic control status, BP control status, and all-cause mortality). *PHCrank_m_*, *X_i_*, *T_m_*, and *u_j_* were the same as in equation (3). The regressions were conducted in the entire sample, the subsample of diabetes patients, and the subsample of hypertension patients. Regression for health outcomes was conducted among the patients who had FBG and BP records in 2023. Patients with missing FBG or BP data were excluded from the regression analysis.

 In addition, we analyzed the impact of management intensity on patients in the high-frequency group and the low-frequency group, respectively.

 All these data analysis was performed using Stata MP 17.^27^

####  Instrumental Variable Regression Model

 As a sensitivity analysis and to mitigate potential reverse causality, the instrumental variable regression model was employed to examine the relationship between the length of follow-up and patients’ healthcare utilization and health outcomes. Moreover, through the utilization of the instrumental variable, the endogenous portion of the independent variable can be eliminated, thereby severing the reverse causal pathway from dependent variables to independent variables.

 The township management intensity (*PHCrank_m_*) was used as an instrumental variable for an individual’s follow-up length:


(5) $$Y_{im}=\beta_{0}+\beta_{1}PHClength_{i}+X_{i}\beta_{2}+T_{m}\beta_{3}+\varepsilon_{i}$$

where *PHClength_i_* was the length of follow-up that patient *i* received. *X_i_*, *T_m_*, and *Y_im_* were the same as in equations (3) and (4). For continuous dependent variables, the two-stage least squares was employed for instrumental variable regression, while for binary dependent variables, an instrumental variable probit model was adopted.

## Results

###  Overview of Patients in Cohort

 Between 2009 and 2023, a total of 60 885 patients in Yuhuan were enrolled in the chronic disease management program. This cohort comprised 21 321 patients under diabetes management and 56 198 patients under hypertension management. For the entire sample, the average age was 68.17, 54.0% were female, 93.7% received at least four follow-ups per year, the average education level was 4.48 years, the average duration since diagnosis was 10.16 years, and the average length of follow-up was 7.61 years.

 Regarding healthcare utilization in 2023, the average number of outpatient visits per patient was 11.59 in PHC settings and 5.12 in hospitals. 5.1% and 17.7% of the patients had inpatient visits in PHC institutions and hospitals, respectively. Total average outpatient expenses per patient in 2023 were 2676.54 RMB (US$379.83), with 1225.20 RMB (US$173.87) spent in PHC settings and 1451.34 RMB (US$205.96) in hospitals. The average inpatient expenses incurred by patients in 2023 were 10 072.68 RMB (US$1429.42), of which 843.51 RMB (US$119.70) was for PHC inpatient visits and 9229.17 RMB (US$1309.72) was for hospital inpatient visits. The average annual inpatient expenses were calculated among patients who were admitted as inpatients in 2023.

 In terms of health outcomes in 2023, 71.0% of patients achieved blood glucose control in the entire sample, with an average FBG level of 6.48 mmol/L. However, for the subsample of diabetes patients, only 49.2% attained glycemic control, with an average FBG of 7.41 mmol/L. 61.8% of patients had their BP under control in the entire sample, with an average SBP of 137.54 mm Hg and an average DBP of 78.88 mm Hg (Table).

**Table:** Characteristics of Patients in the Diabetes or Hypertension Management Program in 2023

	**Entire Sample**		**Subsample of Diabetes Patients**		**Subsample of Hypertension Patients**	
	**Mean/Number**	**SD/Proportion**	**Mean/Number**	**SD/Proportion**	**Mean/Number**	**SD/Proportion**
**Individual characteristic**						
Age	68.17	11.10	67.14	10.41	68.69	11.03
Gender^#^						
Male	27 985	45.9%	9690	45.4%	25 692	45.7%
Female	32 900	54.0%	11 631	54.5%	30 506	54.2%
Years of education	4.48	4.04	4.59	4.08	4.40	4.03
Years since diagnosis	10.16	7.10	10.26	7.35	10.40	7.11
Length of follow-up	7.61	4.50	8.34	4.56	7.74	4.44
**Healthcare utilization**						
Outpatient visits	16.71	12.24	18.78	12.24	16.68	12.32
PHC outpatient visits	11.59	8.96	12.55	9.11	11.64	9.00
Hospital outpatient visits	5.12	8.44	6.23	9.00	5.04	8.47
Inpatient admission^#^						
Not being admitted as inpatients	47 909	78.7%	15 835	74.3%	44 155	78.6%
Being admitted as inpatients	12 976	21.3%	5486	25.7%	12 043	21.4%
PHC inpatient admission^#^						
Not being admitted as PHC inpatients	57 776	94.9%	19 798	92.9%	53 283	94.8%
Being admitted as PHC inpatients	3109	5.1%	1523	7.1%	2915	5.2%
Hospital inpatient admission^#^						
Not being admitted as hospital inpatients	50 138	82.4%	16 903	79.3%	46 254	82.3%
Being admitted as hospital inpatients	10 747	17.7%	4418	20.7%	9944	17.7%
Outpatient expenses	2676.54	3720.53	3431.37	4039.09	2636.20	3753.94
PHC outpatient expenses	1225.20	1350.74	1512.58	1509.02	1215.12	1345.90
Hospital outpatient expenses	1451.34	3443.81	1918.79	3830.85	1421.08	3474.17
Inpatient expenses	10 072.68	15 484.52	10 224.84	16 359.28	10 156.24	15 752.45
PHC inpatient expenses	843.51	2386.09	866.03	2354.75	855.51	2408.08
Hospital inpatient expenses	9229.17	15 572.71	9358.81	16 454.50	9300.73	15 841.61
**Health outcomes**						
FBG	6.48	1.87	7.41	2.02	6.34	1.79
Glycemic control status^#^						
FBG ≥7.0 mmol/L	9808	29.0%	9071	50.8%	7761	25.7%
FBG <7.0 mmol/L	24 058	71.0%	8787	49.2%	22 460	74.3%
SBP	137.54	9.37	138.13	10.16	138.09	9.23
DBP	78.88	6.05	79.07	6.04	79.03	6.09
BP control status^#^						
SBP ≥140 mm Hg or DBP ≥90 mm Hg	18 793	38.2%	7395	41.2%	18 490	40.3%
SBP <140 mm Hg & DBP <90 mm Hg	30 341	61.8%	10 537	58.8%	27 446	59.8%

Abbreviations: SD, standard deviation; FBG, fasting blood glucose; SBP, systolic blood pressure; DBP, diastolic blood pressure; PHC, primary healthcare; BP, blood pressure. Notes: This table showed the individual characteristics, healthcare utilization, and health outcomes of patients in 2023. For dichotomous variables, the number and proportion of patients were employed for descriptive analyses. Dichotomous variables were marked with a ‘#’ in the upper right corner. For continuous variables, the mean and standard deviation were used for descriptive analyses. The mean values of inpatient expenses were calculated among patients who were admitted as inpatients during 2023.

###  Factors Influencing Length of Follow-up and Management Intensity

 Based on the estimates from equation (1), older age and earlier diagnosis were associated with greater length of follow-up, while female patients and those with higher levels of education had a shorter duration of management. At the township level, the number of PHC physicians was negatively correlated with the length of follow-up (Table S1, Supplementary file 1). The variance inflation factor (VIF) was employed to test for multicollinearity. The mean VIF was 1.76 (<5), indicating no substantial multicollinearity concerns (Table S2, Supplementary file 1).

**Figure 3:**
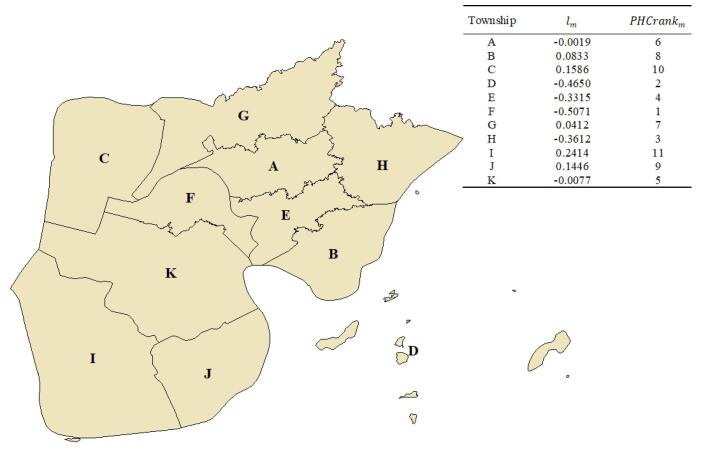
Map of Yuhuan and management intensity of the 11 townships. Notes: This figure demonstrated the geographical location, management intensity scores (*l_m_*), and *PHCrank_m_* for the 11 townships in Yuhuan. *l_m_* and *PHCrank_m_* were calculated from the residual values of equation (1).

 The management intensity score, calculated from the residuals of equation (1), was highest for Township I at 0.2414 and lowest for Township F at -0.5071 in 2023. Therefore, Township I had the highest management intensity with a *PHCrank_m_* of 11, while Township F showed the lowest management intensity with a *PHCrank_m_* of 1 (Figure 3).

###  Impacts of Management Intensity on Patients’ Healthcare Utilization and Expenses

 For the entire sample, each unit increase in the township’s *PHCrank_m_* was associated with an increase of 0.21 PHC outpatient visits per individual (*P*= .001), which was about 1.8% of the average PHC outpatient visits. Conversely, each unit increase in management intensity was linked to a reduction of 0.15 hospital outpatient visits (*P*< .001), equating to 2.9% of the average hospital outpatient visits.

**Figure 4:**
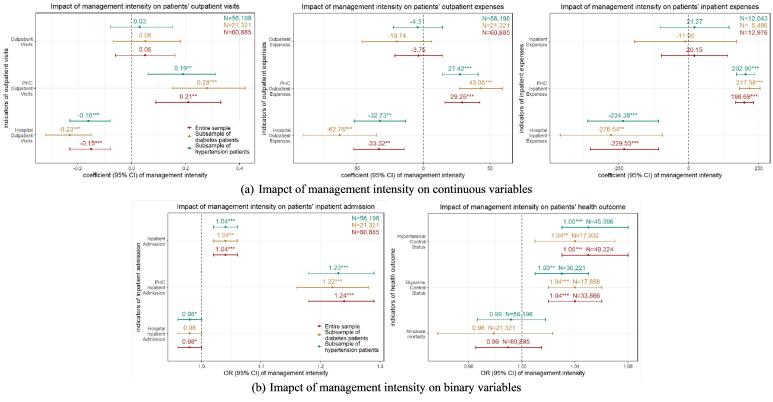
Impact of Management Intensity on Patients’ Healthcare Utilization and Health Outcome. Notes: This figure showed the impact of management intensity on patients. Each estimated point and error bar represented a regression. The estimates for continuous outcome variables were *β_1_* in equation (3), while the estimates for binary outcome variables were the $OR\;(e^{\beta_{1}})$ in equation (4), representing how individuals’ healthcare utilization outcomes varied with *PHCrank_m_*. Abbreviations: PHC, primary healthcare; CI, confidence level; OR, odds ratio.

 Patients with higher management intensity were more likely to have PHC inpatient admission (odds ratio [OR]: 1.24, 95% CI: 1.18-1.29; *P*< .001), and less likely to be an inpatient in hospitals (OR: 0.98, 95% CI: 0.97-1.00, *P*= .026). Furthermore, each unit of increase in the *PHCrank*_m_ was connected with an increase of 198.69 RMB (US$28.20) in an individual’s annual PHC inpatient expenses and a decrease of 229.55 RMB (US$32.58) in a patient’s annual hospital inpatient expenses (Figure 4). It indicated that patients in townships with higher management intensity were more inclined to seek care at PHC institutions and made less utilization of hospital services.

**Figure 5:**
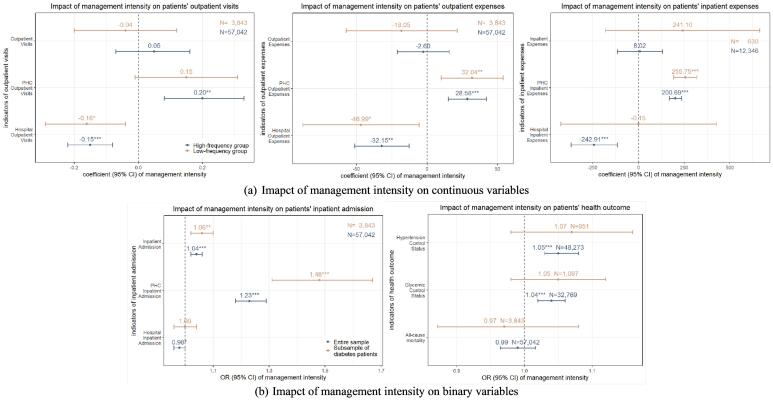
Impact of Management Intensity on Patients’ Healthcare Utilization and Health Outcome (High-Frequency Group and Low-Frequency Group). Notes: This figure showed the impact of management intensity on patients with follow-up ≥4 times/year (high-frequency group) and those with follow-up <4 times/year (low-frequency group). Each estimated point and error bar represented a regression. The estimates for continuous outcome variables were *β_1_* in equation (3), while the estimates for binary outcome variables were the $OR\;(e^{\beta_{1}})$ in equation (4), representing how individuals’ healthcare utilization and health outcomes varied with *PHCrank_m_*. Abbreviations: PHC, primary healthcare; CI, confidence level; OR, odds ratio.

 The regression conducted within the high-frequency group showed similar results. Unlike the results of regression within the entire sample and high-frequency group, the effects of management intensity on PHC outpatient visits and hospital inpatient expenses were statistically insignificant within the low-frequency group (Figure 5).

###  Impacts of Management Intensity on Patients’ Health Outcomes

 The results show that higher management intensity was associated with improved health outcomes among patients. For each increase in *PHCrank_m_* of townships, an individual in the entire sample was more likely to have their blood glucose (OR: 1.04, 95% CI: 1.02-1.06, *P*< .001) and BP (OR: 1.05, 95% CI: 1.03-1.08, *P*< .001) under control. However, there was no significant association between management intensity and all-cause mortality (OR: 0.99, 95% CI: 0.97-1.01, *P*= .417). The results for both the subsample of diabetes patients and the subsample of hypertensive patients were similar to those described above (Figure 4). This indicated that patients in townships with higher management intensity were more likely to maintain effective control over their blood glucose and BP levels.

 The regression conducted within the high-frequency group showed similar results, while the results of the regression conducted within the low-frequency group showed that management intensity did not exert a significant influence on patients’ health outcomes (Figure 5).

###  Results of Sensitivity Analysis

 The results obtained from the instrumental variables method were consistent with the main analysis. Therefore, the estimation results in this study were generally robust.

 In the entire sample, for each additional year in length of follow-up, the number of PHC outpatient visits increased by 2.19, the number of hospital outpatient visits decreased by 2.03, the probability of PHC hospitalization rose by 175%, whereas the probability of hospital hospitalization declined by 13%, the probability of achieving blood glucose control improved by 35%, and the probability of achieving BP control rosed by 29% (Figures S2 and S3, Supplementary file 1).

## Discussion

 This study measured management intensity based on patients’ length of follow-up and highlighted the pivotal role of management intensity in improving the effectiveness of chronic disease management programs in China. Increased management intensity was associated with greater utilization of PHC services, reduced outpatient expenses at hospitals, and improved glycemic and BP control among patients. These findings were consistent with previous research.^15,17,18^ In addition, our study underscored the critical role of PHC physicians’ efforts, supported by the health system and community, in optimizing chronic disease management outcomes.

###  Higher Management Intensity Attracted More Patients to Primary Healthcare Institutions

 Management intensity was relevant to the transition of patients’ healthcare-seeking behaviors from hospital-based care to PHC institutions.

 Higher management intensity may indicate a closer physician-patient relationship, leading to increased patients’ utilization of PHC services. In townships with higher management intensity, PHC physicians dedicated increased effort to providing comprehensive care, including medication guidance, dietary advice, and mental health support. For elderly patients with restricted mobility, they also offered home visits for consultation and rehabilitation. This multifaceted concern and careful interaction could improve patients’ trust in PHC physicians, which was an important step in developing physician-patient relationships and interactions.^28,29^ As the relationships strengthened, patients shifted their visits from hospitals to PHC institutions. Despite increased utilization of PHC inpatient services, there was a significant decrease in hospital inpatient utilization. It suggested that the demand was diverted from hospitals to PHCs with no overall overuse. This trend aligned with the policy direction in China, which prioritized primary care by increasing investments in expanding human resources and upgrading service capacity, aimed to guide patients towards PHC institutions.^30^

 Furthermore, China’s lack of a gatekeeper system in healthcare has complicated efforts to direct residents to PHC institutions.^31^ Enhanced management intensity could improve the responsiveness and attractiveness of PHC institutions, thereby supporting the government’s concerted efforts to establish an integrated healthcare delivery system that prioritized PHC services.

###  The Impact of Management Intensity on Health Outcomes Necessitated Amplification

 Increased management intensity was associated with improved glycemic and BP control. However, compared to other research, the observed improvement remained limited.^32,33^ Therefore, the multi-sector engagement from PHC physicians, the health system, and the community on chronic disease management needs to be enhanced to generate a greater effect size and achieve further health improvements.

 However, the effect of management intensity on glycemic and BP control was not observed in the low-frequency group. Infrequent follow-ups likely hindered PHC physicians from delivering effective services, including timely health assessments and medication adjustments. Such sporadic healthcare service may be insufficient to yield statistically significant clinical benefits for patients.^34^ Therefore, the related policy in China mandated that PHC physicians should conduct a minimum of four follow-up visits per patient annually.

 Furthermore, there was no significant association between management intensity and patient mortality. This may be attributed to the relatively short observation period, limited to the year 2024, which might be insufficient to detect a significant long-term effect. Additionally, the limited sample size may also reduce the statistical power.

###  Enhancing Multi-Sector Engagement Is Key to Increasing Management Intensity

 Since challenges, including heavy workloads and modest salaries, could diminish physicians’ dedication in the context of an aging population,^35,36^ efficient supports from the health system and community were required to enhance PHC physicians’ motivation and capability in providing services for elderly patients. This multi-sector engagement is crucial in improving management intensity.

 The health system and community in Yuhuan engaged in chronic disease management by providing support like health system information, performance-based bonuses, and community workers.^37^ Firstly, the Health Map, an integrated health information system in Yuhuan, supplied PHC physicians with access to historical medical records and health examination information, thereby conserving the effort required for patient consultations. Secondly, the decision support system based on national clinical guidelines and artificial intelligence could generate personalized treatment plans according to patient information. This system provided substantial support for PHC physicians in managing the complex health conditions of elderly patients and streamlined their clinical workflow. Thirdly, community workers in Yuhuan served as a bridge between patients and PHC physicians, facilitating physicians’ understanding of elderly patients’ needs while also conveying health advice to the elderly in a clear manner. Moreover, PHC physicians were incentivized by performance-based bonuses, which were calculated according to their workload in chronic disease management and their scores in key performance indicators.

###  Limitations

 This study has several limitations that should be acknowledged.

 Firstly, regarding the study design, a retrospective cohort design was employed rather than a randomized controlled trial. This study aimed to provide policy recommendations for optimizing chronic disease management, which inherently required evidence reflecting real-world effectiveness. However, randomized controlled trial is typically designed to evaluate the efficacy of an intervention—its optimal performance under highly controlled conditions. Thus, an observational study using real-world data was more suitable for addressing the study’s policy-oriented objectives.

 Secondly, owing to limitations in data availability, this study could not incorporate patient non-adherence and interaction quality into the measurement of management intensity. Therefore, follow-up length was employed as a proxy for management intensity.

 Thirdly, owing to the unavailability of data, this study could not encompass patients outside of chronic disease management, which may introduce confounding. However, the sample remained broadly representative, encompassing over 90% of all chronic disease patients in Yuhuan. Furthermore, due to the lack of HbA1c data—a test unavailable at Chinese PHC institutions during follow-up visits—this study used FBG as a substitute. The lack of data on socioeconomic status and household income limited the ability to control for potential confounders. Consequently, the observed associations may not be interpreted as causal relationships.

## Conclusion

 Greater management intensity was associated with increasing utilization of PHC services and yielded improved health outcomes for patients, driven by improved responsiveness of PHC institutions and strengthened physician-patient relationships. Given the essential role PHC physicians play in delivering chronic disease management services, targeted support from the community and health system should be provided to streamline their work and boost their motivation. Incentivizing PHC physicians to intensify management intensity may serve as a transferable strategy for other LMICs facing challenges in adopting a PHC-focused approach to chronic disease control, especially in the context of population aging.

## Disclosure of artificial intelligence (AI) use

 During the preparation of this work, the authors used Grammarly (https://app.grammarly.com/) in order to check and correct grammatical errors, spelling, and punctuation. After using this tool, the authors reviewed and edited the content as needed.

## Ethical issues

 The data used in this study were previously archived documents or records, and no communication was generated between the researchers and the subjects. Ethical approval was not applicable in this study.

## Conflicts of interest

 Authors declare that they have no conflicts of interest.

## Supplementary files


Supplementary file 1 contains Tables S1-S2, Figures S1-S3, and Data-Cleaning Code.
