# Supplementary material for: Improving Primary Healthcare for Elderly Patients: How Chronic Disease Management Intensity Makes a Difference
Source: Int J Health Policy Manag. 2026 Mar 10;15:9152. doi: 10.34172/ijhpm.9152 (PMC13145240; doi:10.34172/ijhpm.9152)
Supplement: Supplementary file 1 — contains Tables S1-S2, Figures S1-S3, and Data-Cleaning Code. [file ijhpm-15-9152-s001.pdf]

Article title: Improving Primary Healthcare for Elderly Patients: How Chronic Disease Management Intensity Makes a Difference

Journal name: International Journal of Health Policy and Management (IJHPM)

Authors' information: Jia Peng[1,2]¶, Di Liang[1,2]¶, Shailendra Prasad[3], Sumit Kane[4], Weijun Zhang[5], Yuxia Wu[6], Jiayan Huang[1,2]*, Yongsong Luo[7], Yin Dong[8]

[1]School of Public Health, Shanghai Institute of Infectious Disease and Biosecurity, Fudan University, Shanghai, China.

[2]NHC Key Laboratory of Health Technology Assessment, Fudan University, Shanghai, China.

[3]Center for Global Health and Social Responsibility, University of Minnesota, Minneapolis, MN, USA. [4]Nossal Institute for Global Health, The University of Melbourne, Melbourne, VIC, Australia.

[5]David Geffen School of Medicine, University of California Los Angeles, Los Angeles, CA, USA.

[6]Yichuan Street Community Health Service Center of Putuo District, Shanghai, China.

[7]Health Commission of Yuhuan, Taizhou, Zhejiang Province, China.

[8]The People's Hospital of Yuhuan, Taizhou, China.

***Correspondence to:** Jiayan Huang; Email: jiayanhuang@fudan.edu.cn

¶Both authors contributed equally to this paper.

Citation: Peng J, Liang D, Prasad S, et al. Improving primary healthcare for elderly patients: how chronic disease management intensity makes a difference. Int J Health Policy Manag. 2026;15:9152. doi:10.34172/ijhpm.9152

Supplementary file 1

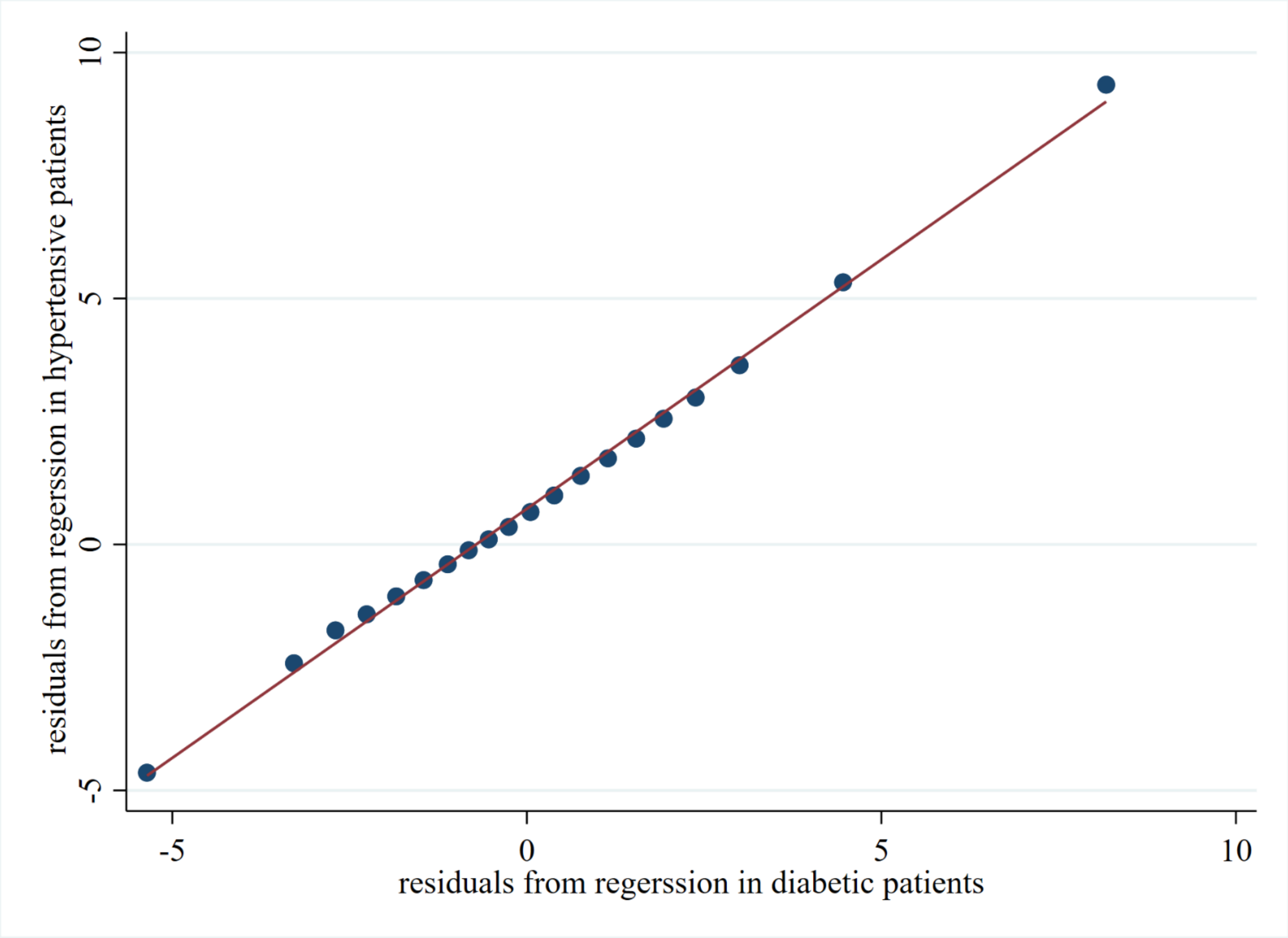

Figure S1. Correlation of management intensity calculated in diabetes sample with that calculated in hypertension sample

Notes: This figure is a binscatter plot of management intensity calculated in the diabetes sample against that calculated in the hypertension sample. The horizontal coordinate was the residual value (η_i) at the individual level obtained by regression in the diabetes sample. The vertical coordinate was the residual value (η_i) at the individual level obtained by regression in the hypertensive sample.

Table S1. Factors affecting length of follow-up

	coefficient (95% CI)	t	P value
Age	0·01 (0·01, 0·02)	15·23	<0·001
gender (reference: male)			
Female	-0·07 (-0·12, -0·03)	-3·59	<0·001
years of education	-0·02 (-0·03, -0·02)	-8·13	<0·001
years since diagnosis	0·80 (0·79, 0·80)	301·23	<0·001
age squared	-0·01 (-0·01, -0·01)	-10·58	<0·001
years since diagnosis squared	-0·01 (-0·01, -0·01)	-145·72	<0·001
number of PHC physicians	-0·22 (-0·27, -0·16)	-7·72	<0·001
being enrolled in diabetes management or not (reference: not)			
patients under diabetes management	1·33 (1·29, 1·37)	60·64	<0·001
being enrolled in hypertension management or not (reference: not)			
patients under hypertension management	0·91 (0·83, 0·98)	22·82	<0·001
Constant	-0·59 (-0·75, -0·42)	-7·02	<0·001

Notes: Appendix table 1 showed the results of equation (1), and the number of observations was 60,885. The dependent variable of this regression was the length of follow-up (number of years) for patients, and the independent variables were listed above in the table. This regression (ordinary least square) covered all the patients who were enrolled in diabetes or hypertension management from 2009 to 2023 in Yuhuan. The coefficients in this table represented how patients' length of follow-up varied with the independent variables.

Table S2. Variance inflation factor of independent variables

Variable	VIF
Age	1.17
Gender	1.37
years of education	1.41
years since diagnosis	3.84
age squared	1.02
years since diagnosis squared	3.63
number of PHC physicians	1.01
being enrolled in diabetes management or not	1.2
being enrolled in hypertension management or not	1.22
Mean VIF	1.76

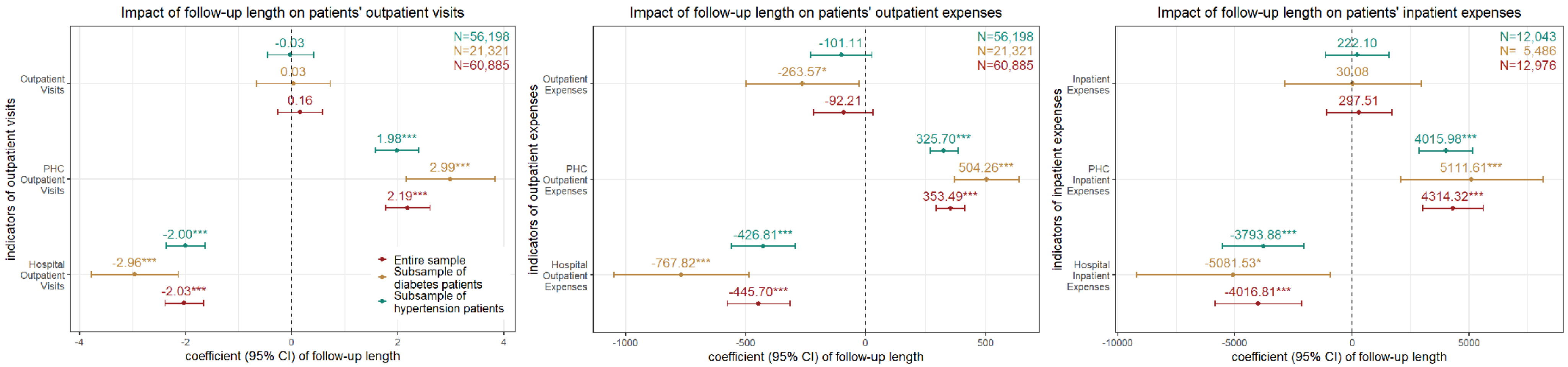

(a) Imapct of follow-up length on continuous variables

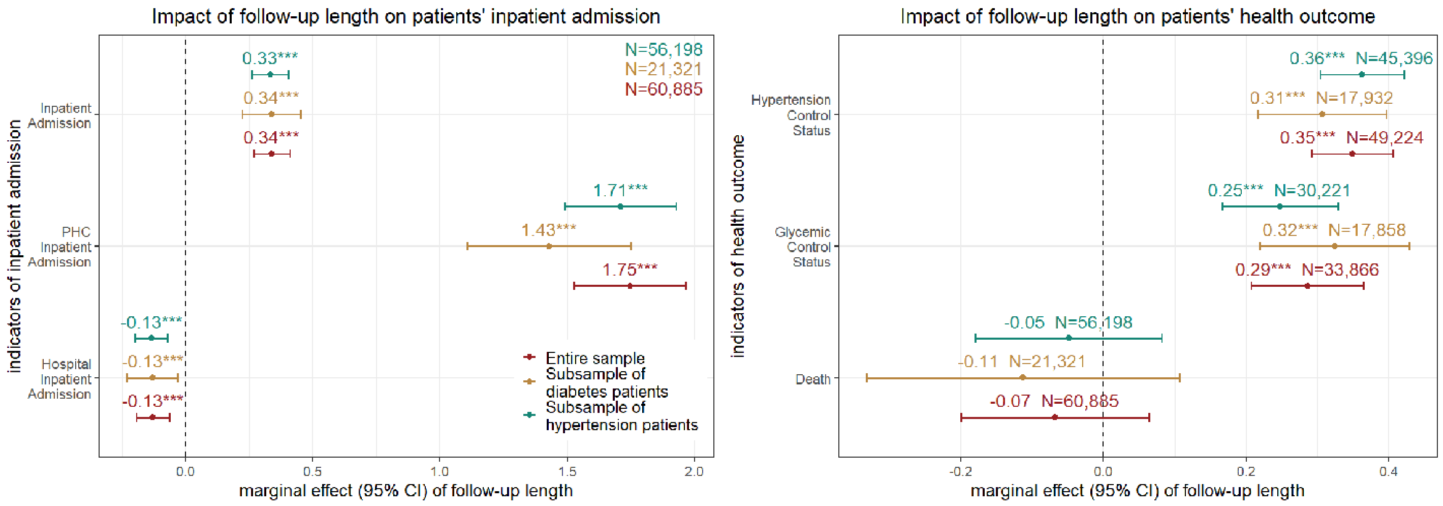

(b) Imapct of follow-up length on binary variables

Figure S2. Impact of management intensity on patients' healthcare utilization and health outcome—results using instrumental variable

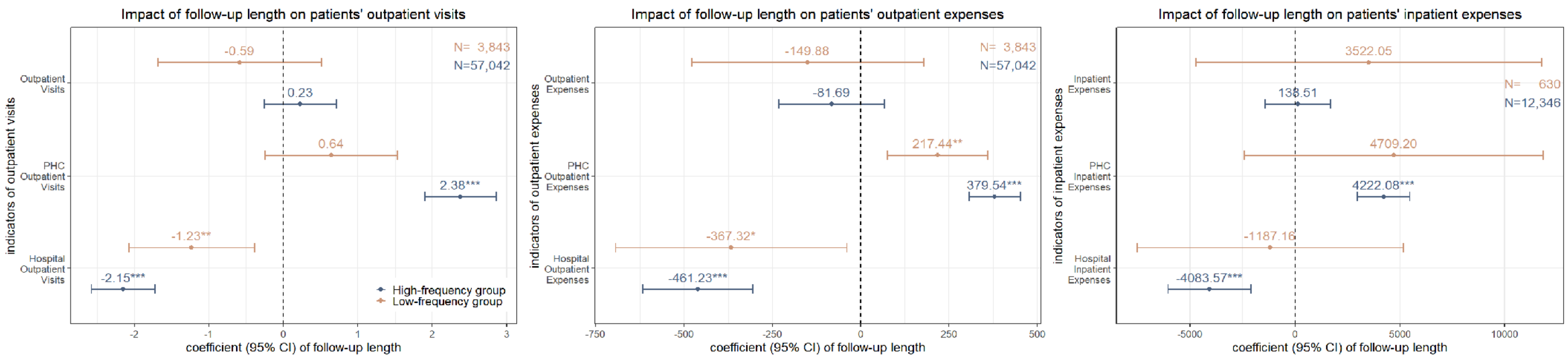

(a) Imapct of follow-up length on continuous variables

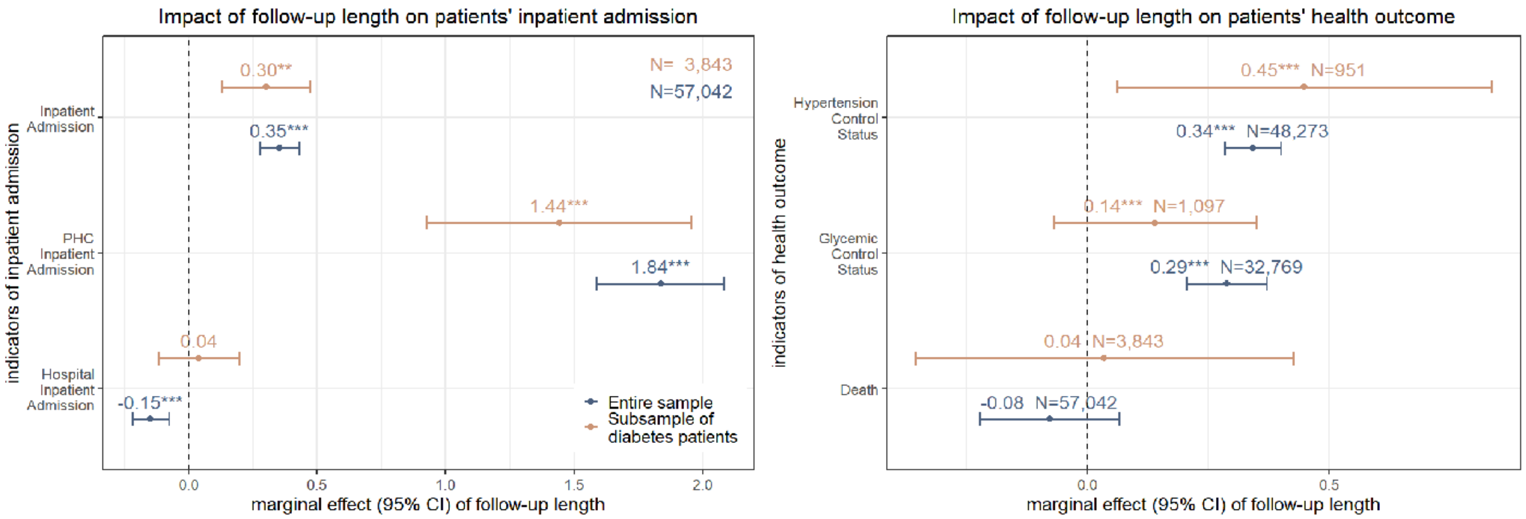

(b) Imapct of follow-up length on binary variables

Figure S3. Impact of management intensity on patients' healthcare utilization and health outcome (high-frequency group and low-frequency group) —results using instrumental variable

Data-cleaning code

```
*1.cleaning process of Electronic Medical Records
keep if year==2023
drop if missing(ID)    // 5.81 million records remained

gen PHC_outpatient_visits=1 if visit_type="PHC"
replace PHC_outpatient_visits=0 if missing(PHC_outpatient_visits)
gen hospital_outpatient_visits=1 if visit_type="hospital"
replace hospital_outpatient_visits=0 if missing(hospital_outpatient_visits)
gen outpatient_visits = PHC_outpatient_visits + hospital_outpatient_visits
collapse (sum) outpatient_visits PHC_outpatient_visits hospital_outpatient_visits, by(ID)
save "C:\Users\26573\Desktop\Records of outpatient visits.dta"
//Calculate the number of outpatient visits for each patient in 2023

gen PHC_inpatient_admission=1 if visit_type="PHC"
replace PHC_inpatient_admission=0 if missing(PHC_inpatient_admission)
gen hospital_inpatient_admission=1 if visit_type="hospital"
replace hospital_inpatient_admission=0 if missing(hospital_inpatient_admission)
gen inpatient_admission = PHC_inpatient_admission + hospital_inpatient_admission
collapse (sum) inpatient_admission PHC_inpatient_admission hospital_inpatient_admission, by(ID)
replace inpatient_admission=1 if inpatient_admission>1
replace PHC_inpatient_admission=1 if PHC_inpatient_admission>1
replace hospital_inpatient_admission=1 if hospital_inpatient_admission>1
save "C:\Users\26573\Desktop\Records of inpatient admission.dta"
//Establish indicators if inpatient admission (=1 if admitted)

replace PHC_outpatient_expenses=0 if missing(PHC_outpatient_expenses)
replace hospital_outpatient_expenses=0 if missing(hospital_outpatient_expenses)
gen outpatient_expenses = PHC_outpatient_expenses + hospital_outpatient_expenses
collapse (sum) outpatient_expenses PHC_outpatient_expenses hospital_outpatient_expenses, by(ID)
save "C:\Users\26573\Desktop\Records of outpatient expenses.dta"
//Calculate outpatient expenses for each patient in 2023

replace PHC_inpatient_expenses=0 if missing(PHC_inpatient_expenses)
replace hospital_inpatient_expenses=0 if missing(hospital_inpatient_expenses)
gen inpatient_expenses = PHC_inpatient_expenses + hospital_inpatient_expenses
collapse (sum) inpatient_expenses PHC_inpatient_expenses hospital_inpatient_expenses, by(ID)
save "C:\Users\26573\Desktop\Records of inpatient expenses.dta"
//Calculate inpatient expenses for each patient in 2023



*2.cleaning process of Death Registration Information
keep if year==2024     // 2,776 records remained
```

```
gen All-cause_mortality=1



*3.cleaning process of Chronic Disease Management Registration
duplicates drop ID, force   // 60,918 records remained
drop if missing(institution)  //60,885 records remained



*4.cleaning process of Service Records of Follow-up Care
cd C:\Users\26573\Desktop
use "Service Records of Follow-up Care.dta", clear
merge 1:1 ID using "Records of Health Check-ups.dta" // Supplement of records form Health Check-ups
keep if _m==3
drop merge  //0.04 million records matched, 0.83 million records in total
egen std_FBG = sd(FBG)
egen mean_FBG = mean(FBG)
drop if FBG < mean_FBG-3*std_FBG | FBG > mean_FBG+3*std_FBG
/*Calculate the mean and standard deviation of fasting blood glucose.
Remove outliers based on the 3σ criterion.*/
egen std_SBP = sd(SBP)
egen mean_SBP = mean(SBP)
drop if SBP < mean_SBP-3*std_SBP | SBP > mean_SBP+3*std_SBP
/*Calculate the mean and standard deviation of systolic blood pressure.
Remove outliers based on the 3σ criterion.*/
egen std_DBP = sd(DBP)
egen mean_DBP = mean(DBP)
drop if DBP < mean_DBP-3*std_DBP | DBP > mean_DBP+3*std_DBP
/*Calculate the mean and standard deviation of diastolic blood pressure.
Remove outliers based on the 3σ criterion.*/
collapse (mean) FBG SBP DBP, by(ID)
save "C:\Users\26573\Desktop\Service Records of Follow-up Care.dta", replace



/*5.the linkage of the four databases
Link the other three databases to the Chronic Disease Management Registration
database using the patient's ID.*/
cd C:\Users\26573\Desktop
use "Service Records of Follow-up Care.dta", clear
merge 1:1 ID using "Records of outpatient visits.dta"
keep if _m==3
drop merge
merge 1:1 ID using "Records of inpatient admission.dta"
```

```
keep if _m==3
drop merge
merge 1:1 ID using "Records of outpatient expenses.dta"
keep if _m==3
drop merge
merge 1:1 ID using "Records of inpatient expenses.dta"
keep if _m==3
drop merge
//Link Electronic Medical Records with Chronic Disease Management Registration

merge 1:1 ID using "Death Registration Information.dta"
keep if _m==3
drop merge
//Link Death Registration Information with Chronic Disease Management Registration

merge 1:1 ID using "Service Records of Follow-up Care.dta"
keep if _m==3
drop merge
//Link Service Records of Follow-up Care with Chronic Disease Management Registration
```
